# Assessment of Home Exercise Adherence in Patients with Non-Surgical Post-Traumatic Neck Pain: A Study Protocol Using the Exercise Adherence Rating Scale

**DOI:** 10.3390/healthcare14121728

**Published:** 2026-06-16

**Authors:** José Antonio Martínez Fernández, Luis Socarrás-Alonso, Daniel Pabón Carrasco

**Affiliations:** 1 Department of Physiotherapy, University of Seville, 41009 Seville, Spain; jamartinez@us.es; 2 Department of Human Anatomy and Embryology, Faculty of Medicine, University of Seville, 41009 Seville, Spain; lsocarras@us.es

**Keywords:** treatment adherence and compliance, exercise therapy, neck pain, whiplash injuries, rehabilitation, physical therapy modalities, patient-reported outcome measures, home exercise, physiotherapy

## Abstract

**Highlights:**

**What are the study objectives and methodological features?**

- This protocol establishes a standardised framework to assess self-reported home exercise adherence in adults undergoing rehabilitation for whiplash-associated disorders or non-surgical post-traumatic neck pain.
- It predefines the targeted population, eligibility criteria, timing of the Exercise Adherence Rating Scale (EARS) administration, core variables, and a structured statistical analysis plan.

**What are the potential methodological contributions?**

- The protocol aims to support consistent and reproducible documentation of adherence within cervical trauma rehabilitation settings.
- It provides a methodological basis to help researchers and clinicians systematically document exercise exposure, facilitating clearer distinctions between treatment efficacy and adherence issues.

**Abstract:**

**Background/Objectives:** Whiplash-associated disorders (WADs) and non-surgical post-traumatic neck pain often require active rehabilitation, including prescribed home exercise, where poor adherence can limit treatment effectiveness. This protocol describes a standardised observational method to assess home exercise adherence using the Exercise Adherence Rating Scale (EARS). **Methods:** This prospective protocol outlines eligibility criteria, recruitment settings, core variables, and a structured EARS administration procedure four weeks post-prescription. The primary outcome is the six-item EARS adherence section score. A target sample of approximately 75 participants will be recruited to ensure at least 62 analyzable participants. **Outputs:** The protocol will generate descriptive data on adherence levels, questionnaire completion rates, and missing data to evaluate implementation feasibility. Planned analyses testing potential associations between adherence and clinical variables will be strictly exploratory and hypothesis-generating. **Conclusions:** This study operationalizes a standardised method for tracking self-reported adherence in cervical trauma rehabilitation, enhancing methodological transparency and reproducibility.

## 1. Introduction

Whiplash-associated disorders (WADs) occur after acceleration–deceleration mechanisms affecting the neck and may lead to neck pain, stiffness, restricted movement, headache, dizziness, upper-limb symptoms, and functional limitations [1,2]. The available epidemiological evidence indicates that WAD after traffic collisions affects a relevant proportion of the population and represents a considerable health, social, and economic burden [1,3]. Although many patients recover during the first months after injury, recovery is not uniform. A best-evidence synthesis from the Bone and Joint Decade Task Force reported that approximately 50% of people with WADs continue to report neck pain one year after injury [2]. Distinct clinical trajectories have also been described, including chronic-severe pain/disability and persistent post-traumatic stress symptoms [4].

Usual conservative care for WADs and non-surgical post-traumatic neck pain typically includes reassurance, education, advice to remain active, gradual return to usual activities, supervised physiotherapy when indicated, and therapeutic exercise delivered both in clinic and at home [5,6]. Current clinical practice guidelines recommend multimodal management combining education, self-management advice, exercise therapy, mobilisation or manual therapy, and functional progression according to the patient’s clinical profile [5,6]. Systematic reviews support the use of active interventions and exercise therapy, although the quality of evidence remains variable, and no single exercise approach has been consistently shown to be superior for all patients [7,8,9].

The effectiveness of exercise-based rehabilitation depends not only on what is prescribed, but also on the degree to which patients implement the prescribed programme. Treatment adherence is not passive obedience to clinical instructions, but an active process in which the patient’s behaviour corresponds with agreed recommendations from the healthcare professional [10,11]. In physiotherapy, adherence is multidimensional and may include attendance at treatment sessions, active participation during treatment, performance of home exercise, frequency and quality of execution, and avoidance of contraindicated activities [12].

Home exercise adherence is particularly challenging because the programme is performed outside the supervised clinical environment and depends on self-efficacy, motivation, social support, symptom behaviour, contextual barriers, and the feasibility of integrating exercise into daily life [13,14,15,16,17,18,19]. In cervical trauma rehabilitation, adherence assessment is especially relevant because symptoms may fluctuate over time, psychological factors may influence recovery, and home exercise is often used to promote self-management and progressive return to function. Without standardised adherence assessment, it is difficult to determine whether limited improvement reflects treatment ineffectiveness or insufficient exposure to the prescribed programme.

Measuring adherence to home exercise remains a methodological problem. Systematic reviews have shown marked heterogeneity in the methods used to assess adherence in home-based rehabilitation, with frequent reliance on diaries, self-reported questionnaires, and ad hoc measures [20,21,22]. This lack of standardisation limits comparability between studies and reduces the interpretability of exercise-based rehabilitation outcomes.

The Exercise Adherence Rating Scale (EARS) was developed to provide a brief, exercise-specific, patient-reported measure of adherence to prescribed home exercise [23]. The main adherence section consists of six items scored on a Likert-type scale, with a total score ranging from 0 to 24, where higher scores indicate greater adherence [23,24]. The original development study reported a one-factor structure explaining 71% of the variance in exercise adherence and acceptable psychometric properties [23]. Since then, the EARS has been adapted and evaluated in several languages and musculoskeletal populations, including Brazilian Portuguese, Nepali, Japanese, Swedish, Mexican Spanish and Persian versions [24,25,26,27,28,29].

To the authors’ knowledge, no previous protocol has specifically standardised the use of the EARS to assess adherence to prescribed home exercise in adults undergoing rehabilitation after WAD or non-surgical post-traumatic neck pain. This does not imply that the EARS has been psychometrically validated for this population. Rather, it identifies the need for a reproducible implementation protocol that can support consistent adherence assessment and inform future validation studies.

The primary research question of this protocol is as follows: What is the level of self-reported adherence to prescribed home exercise, measured with the EARS, in adults undergoing rehabilitation after WAD or non-surgical post-traumatic neck pain? Secondary research questions are as follows: Is EARS administration feasible in this clinical context, and are EARS scores associated with predefined clinical or rehabilitation-related variables such as pain intensity, time since injury, number of supervised sessions, and characteristics of the prescribed home exercise programme? The exploratory hypothesis is that lower adherence may be associated with higher pain intensity, longer time since injury, lower supervised contact, and higher prescribed home exercise burden. Publishing this study protocol prior to data acquisition promotes scientific transparency and reproducibility. By pre-registering the study hypotheses, eligibility criteria, and primary endpoints, this framework mitigates publication bias and prevents selective outcome reporting. Furthermore, early dissemination allows independent investigators to evaluate or replicate the tracking infrastructure, contributing to the standardisation of exercise adherence metrics within cervical trauma rehabilitation before data collection begins.

## 2. Experimental Design

### 2.1. Study Design

This manuscript describes a prospective observational protocol for assessing self-reported adherence to prescribed home exercise. It does not propose an experimental intervention, randomisation, or allocation to treatment groups. The protocol is designed to standardise adherence assessment in patients receiving usual physiotherapy care after WAD or non-surgical post-traumatic neck pain.

The protocol does not aim to evaluate the therapeutic effectiveness of a specific physiotherapy intervention and does not aim to validate the EARS psychometrically in this population. Psychometric validation, including reliability, validity, responsiveness, and floor or ceiling effects, is considered a subsequent research step. The present protocol focuses on standardised implementation, feasibility of administration, and exploratory associations between EARS scores and predefined contextual variables.

### 2.2. Setting and Recruitment

Participants will be recruited from outpatient physiotherapy and rehabilitation clinics affiliated with the University of Seville or collaborating clinical centres. Recruitment will begin only after approval by the corresponding Ethics Committee and is expected to continue for approximately 12 months, or until the target sample size has been reached.

### 2.3. Target Population

The target population will consist of adults receiving conservative physiotherapy for WAD grades I–II or non-surgical post-traumatic neck pain without fracture, instability, or other red-flag conditions. To reduce clinical heterogeneity, the protocol will not include patients requiring surgical management, urgent medical management, or specialised neurological care. Time since injury will be recorded and categorised a priori as early/subacute (up to 3 months), persistent (more than 3 to 6 months), or chronic (more than 6 months), allowing clinical context to be considered in exploratory analyses.

### 2.4. Eligibility Criteria

#### 2.4.1. Inclusion Criteria

Participants must meet the following criteria:

1. Age 18 years or older.
2. Diagnosis of WAD grades I-II or non-surgical post-traumatic neck pain.
3. Referral to physiotherapy or rehabilitation after cervical trauma.
4. Prescription of a home exercise programme as part of usual rehabilitation.
5. Ability to understand the participant information sheet and complete the questionnaire.
6. Written informed consent before data collection.

#### 2.4.2. Exclusion Criteria

Participants will be excluded if they present any of the following:

1. Severe cognitive impairment or inability to complete self-reported questionnaires.
2. Insufficient comprehension of the questionnaire language.
3. Severe neurological deficit requiring urgent medical management.
4. Cervical fracture, instability, infection, tumour, or another red-flag condition requiring medical or surgical management rather than standard outpatient physiotherapy.
5. Refusal or inability to provide informed consent.

### 2.5. Home Exercise Programme Documentation and Standardisation

Because adherence can only be interpreted in relation to a known prescription, all home exercise programmes must be documented before EARS administration. The protocol does not require all patients to receive the same exercises, because programmes are expected to reflect usual individualised care. However, a minimum set of prescription characteristics will be mandatory to ensure reproducibility and allow comparison across participants.

A home exercise programme will be eligible for adherence assessment only if it includes a clearly defined list of exercises, prescribed weekly frequency, estimated session duration, recommended number of sets and repetitions or duration per exercise when applicable, and any progression instructions. The method of patient instruction will also be recorded, including verbal explanation, therapist demonstration, written handout, video material, digital platform, app-based support, or self-monitoring tools. Any sensory or communication adaptations required for older adults or participants with visual or hearing limitations will be documented.

### 2.6. Protocol Variables

#### 2.6.1. Primary Variable

The primary outcome will be adherence to prescribed home exercise, assessed using the total score of the six-item EARS adherence section four weeks after prescription of the home exercise programme [23]. The EARS total score will be analysed as a continuous variable. No validated cut-off for low or high adherence exists for WADs or non-surgical post-traumatic neck pain; therefore, no diagnostic classification based on EARS cut-off values will be used. Any exploratory categorisation, such as tertiles or sample-based percentiles, will be clearly labelled as exploratory and not clinically validated.

#### 2.6.2. Mandatory Core Variables

The following secondary variables will be collected to contextualise adherence:

- Age and sex.
- Diagnostic category: WAD grade I-II or non-surgical post-traumatic neck pain.
- Time since injury and predefined time-since-injury category.
- Mechanism of injury.
- Pain intensity using a numerical rating scale or visual analogue scale (VAS).
- Number of supervised physiotherapy sessions completed before EARS administration.
- Home exercise programme characteristics: number of exercises, prescribed weekly frequency, estimated session duration, dose, progression instructions, and instructional format.
- EARS total score and item-level missingness.
- Self-perceived barriers to exercise.

#### 2.6.3. Optional Contextual Variables

When available, additional contextual variables will be recorded to supplement the primary database, including medication use, relevant comorbidities, and use of digital self-monitoring tools. These variables will not be required for the primary descriptive analysis but may inform exploratory analyses or future protocol refinement.

### 2.7. Exercise Adherence Rating Scale

The EARS will be administered according to the original publication and the selected Spanish-language adaptation [23,28]. The full scale will not be reproduced in the manuscript or Supplementary Material unless permission is obtained from the relevant rights holder. Researchers or clinicians wishing to use the scale should obtain it from the original publication, the published adaptation selected for their setting, or from the developers or rights holder when required. The protocol will use the six-item main adherence section as the primary measure.

### 2.8. Timing of EARS Administration

The primary EARS assessment will be performed four weeks after the prescription of the home exercise programme. This timing was selected to provide sufficient exposure to the prescribed programme while limiting excessive recall burden. If a participant has not had at least two weeks of opportunity to perform the prescribed home programme because of illness, interruption of care, or other reasons, EARS administration will be postponed until this minimum exposure has occurred, and the delay will be recorded.

### 2.9. Sample-Size Rationale

The sample-size rationale is based on the precision of the descriptive estimate of the mean EARS score rather than on hypothesis testing. Assuming a conservative standard deviation of approximately 6 points for the EARS total score and a desired 95% confidence interval half-width of approximately 1.5 points, at least 62 analyzable participants would be required. To account for incomplete questionnaires, withdrawal, or loss to follow-up, the target recruitment sample will be approximately 75 participants. Exploratory association analyses will be interpreted cautiously and will not be considered confirmatory.

### 2.10. Ethical and Data Protection Considerations

The protocol will be submitted to the corresponding research ethics committee before any data collection begins. No participant recruitment or research data collection will take place before the relevant ethical approval has been obtained. Participation will be voluntary, and patients will receive written and verbal information about the aim of the study, the nature of their participation, the type of data collected, and their right to withdraw without consequences for their clinical care.

Only data necessary for the declared research objective will be collected. Personal identifying data will be separated from the analytical database. Each participant will be assigned a unique alphanumeric code. Access to identifiable data will be restricted to authorised members of the research team. Results will be reported in aggregate form, preventing individual participant identification.

## 3. Materials and Equipment

### 3.1. Participant Information and Consent Document

Draft templates for the participant information sheet, informed consent form, personal data processing consent form, and consent withdrawal form will be provided as Supplementary Material. These documents are draft materials and will require approval by the corresponding Ethics Committee before use in participants.

### 3.2. Clinical Data Collection Sheet

A structured clinical data collection sheet will be used to record the mandatory core variables and optional contextual variables. The form will be linked to the participant code rather than directly to identifiable information.

### 3.3. Home Exercise Programme Record

A structured home exercise programme record will be used to document the prescribed exercises, frequency, dose, duration, progression, and instruction format. This record is essential for interpreting EARS responses because adherence depends on the characteristics of the prescription.

### 3.4. Data Storage System

Data will be stored in a secure digital file or an encrypted database. If paper questionnaires are used, they will be kept in a locked cabinet in a restricted-access area. Digital files will be password-protected and stored according to institutional data protection policies.

### 3.5. Statistical Software

Data will be analysed using IBM SPSS Statistics, version 29.0 (IBM Corp., Armonk, NY, USA), R, or an equivalent statistical package available at the time of analysis. The software and version used will be reported in any subsequent empirical publication.

## 4. Detailed Procedure

### 4.1. Identification of Eligible Participants

Patients attending physiotherapy after WAD or non-surgical post-traumatic neck pain will be screened according to the eligibility criteria by a member of the research or clinical team familiar with the protocol.

### 4.2. Participant Information and Consent

Eligible patients will receive the participant information sheet. The researcher or clinician will explain the study in clear language, clarify that participation is voluntary, and indicate that refusal to participate will not affect clinical care. Patients who agree to participate will sign the informed consent form before any study-specific data are collected.

### 4.3. Participant Code Assignment

After consent, each participant will be assigned a unique alphanumeric code. This code will be used on questionnaires and data collection forms. The correspondence between participant identity and code will be stored separately from the analytical database.

### 4.4. Baseline Clinical Data Collection

Baseline data will be collected using the structured clinical data sheet. The mandatory dataset will include sociodemographic variables, diagnostic category, time since injury, mechanism of injury, pain intensity, and rehabilitation-related variables.

### 4.5. Recording of the Prescribed Home Exercise Programme

The prescribed home exercise programme will be recorded before EARS administration using the structured programme record. If the prescription is insufficiently defined, the clinician will complete the prescription details before the participant is considered eligible for adherence assessment.

### 4.6. EARS Administration

The EARS will be administered four weeks after the prescription of the home exercise programme. Participants should complete the questionnaire independently whenever possible. To mitigate social desirability bias, participants will be informed that there are no right or wrong answers, that responses will not affect their clinical care, and that honest reporting is needed to understand the feasibility of home exercise prescription. The treating clinician should not suggest responses or frame adherence as a desirable behaviour during completion.

### 4.7. Missing-Data Management

Before storing the questionnaire, the researcher will check whether all EARS items have been completed. If any item is missing, the participant may be asked to complete it if still present and willing to do so. If the EARS total score cannot be calculated according to scoring recommendations, the participant will be excluded from the primary EARS analysis but retained in descriptive missing-data reporting.

### 4.8. Data Coding and Anonymisation

Questionnaire scores and clinical variables will be entered into the database using only the participant code. Identifiable information will not be included in the analysis file. Data entry will be reviewed to detect possible transcription errors.

### 4.9. Statistical Analysis

Descriptive statistics will be used to summarise participant characteristics, home exercise prescription characteristics, and EARS scores. Continuous variables will be inspected using histograms, Q-Q plots, and the Shapiro–Wilk test. Normally distributed quantitative variables will be expressed as mean and standard deviation; non-normally distributed variables will be expressed as median and interquartile range. Categorical variables will be expressed as frequencies and percentages.

The primary analysis will describe the EARS total score as a continuous variable with 95% confidence intervals when appropriate. Item-level missingness, completion rate, and time required to complete the questionnaire will be reported as feasibility indicators. Because no validated cut-off for low adherence exists in this population, no confirmatory dichotomisation of EARS scores will be performed.

Exploratory associations between EARS total score and continuous variables such as pain intensity, time since injury, number of supervised sessions, and prescribed home exercise duration will be examined using Pearson or Spearman correlation coefficients, depending on distributional assumptions. Differences in EARS scores across categorical variables such as sex, diagnostic category, time-since-injury category, or instruction format will be explored using t-tests, Mann–Whitney U tests, analysis of variance, or Kruskal–Wallis tests as appropriate.

To examine potential contextual factors influencing adherence, an exploratory multivariable linear regression will be conducted to assess associations between the EARS total score and predefined candidate variables. This multivariable analysis will only be performed if the final data structure is appropriate and a minimum participant-to-variable ratio of 10:1 is satisfied to prevent model overfitting. Candidate variables selected a priori based on clinical relevance include age, sex, pain intensity, time since injury, number of supervised sessions, and estimated weekly home exercise burden. If the final sample size of analyzable participants falls below the required 10:1 ratio, or if core regression assumptions (specifically linearity, homoscedasticity, and the absence of multicollinearity) are violated, the multivariable model will be omitted, and analyses will be limited to bivariate correlations. Any multivariable results will be interpreted strictly as exploratory and hypothesis-generating. Regression assumptions will be checked using residual plots and collinearity diagnostics.

Analyses will be interpreted as exploratory. No formal correction for multiple testing will be applied to exploratory analyses; instead, effect sizes, confidence intervals, and consistency of findings will be emphasised. Missing data will be described. The primary analysis will use available complete EARS total scores; no imputation will be performed for the primary descriptive estimate unless prespecified scoring rules support it. If missing data exceed 10% for key variables, sensitivity analyses will be considered.

### 4.10. Critical Points and Methodological Warnings

The following points are critical for reproducibility:

1. The home exercise programme must be clearly documented before adherence is interpreted.
2. The primary EARS administration time point must be four weeks after prescription or after a recorded minimum exposure period.
3. Participants must complete the questionnaire without clinician influence.
4. Instructions must emphasise that honest responses are needed and that answers will not affect clinical care.
5. The linguistic version of the questionnaire must be appropriate for the target population.
6. Data must be anonymised before analysis.
7. The procedure for managing missing data must be predefined.

Potential sources of bias include recall bias, social desirability, variability in prescribed exercise programmes, and the inability of self-report measures to assess technical quality of exercise execution [20,21,22,23,24,27]. The four-week timing, neutral administration instructions, independent completion, standardised prescription recording, and missing-data procedures are intended to reduce these risks, although they cannot eliminate them.

## 5. Planned Study Outputs and Methodological Objective

This protocol focuses on generating standardised descriptive estimates of self-reported home exercise adherence in adults undergoing rehabilitation after WADs or non-surgical post-traumatic neck pain. The primary planned outputs consist of data regarding the distribution of EARS total scores, questionnaire completion rates, item-level missingness, and the descriptive characteristics of the prescribed home exercise programmes under assessment. Additionally, the study will collect exploratory data on candidate variables to examine potential associations with adherence—specifically pain intensity, time since injury, supervised physiotherapy exposure, and exercise prescription design. These analyses are strictly hypothesis-generating and will not be interpreted as confirmatory evidence. From a methodological perspective, the implementation of this protocol aims to evaluate feasibility outcomes crucial for future research, including the acceptability of EARS administration, the adequacy of the selected Spanish-language version for this target population, the consistency of home exercise documentation, and the logistical feasibility of tracking adherence variables within routine rehabilitation settings.

## Data Availability

The manuscript describes a study protocol and does not report participant-level data. No datasets were generated or analysed during the preparation of this protocol. Future data availability will depend on ethical approval, informed consent conditions, and institutional data protection policies.

## Supplementary Materials

The following supporting information can be downloaded at: https://www.mdpi.com/article/10.3390/healthcare14121728/s1. File S1: Draft participant information sheet; File S2: Draft informed consent form; File S3: Draft personal data processing consent form; File S4: Draft consent withdrawal form; Table S1: Draft clinical data collection sheet; Table S2: Draft home exercise programme record. These documents are draft templates and will require approval by the corresponding Ethics Committee before use. The Exercise Adherence Rating Scale will be administered according to the original publication and the selected Spanish-language adaptation. The full scale is not reproduced in this manuscript or in the supplementary material.
