# Supplementary material for: Assessment of Home Exercise Adherence in Patients with Non-Surgical Post-Traumatic Neck Pain: A Study Protocol Using the Exercise Adherence Rating Scale
Source: Healthcare (Basel). 2026 Jun 16;14(12):1728. doi: 10.3390/healthcare14121728 (PMC13300558; doi:10.3390/healthcare14121728)
Supplement: Supplementary file 1 [file healthcare-14-01728-s001.zip › healthcare-4353462-supplementary.pdf]

# File S1. PARTICIPANT INFORMATION SHEET

**Draft template for manuscript supplementary material. This document must be reviewed and approved by the corresponding Research Ethics Committee before use with participants.**

**Study title:** Assessment of Home Exercise Adherence in Adults with Whiplash-Associated Disorders and Non-Surgical Post-Traumatic Neck Pain: A Protocol Study Using the Exercise Adherence Rating Scale

**Principal investigator:** José Antonio Martínez Fernández

**Contact person:** Daniel Pabón Carrasco (dpabon@us.es)

**Organisation:** University of Seville

**Study site:** Outpatient physiotherapy and rehabilitation clinics affiliated with or collaborating with the University of Seville, Seville, Spain

**Ethics approval:** To be completed after approval by the corresponding Research Ethics Committee

## Information about the study

You are being invited to participate in a research study about adherence to prescribed home exercise in adults receiving physiotherapy after whiplash-associated disorders or non-surgical post-traumatic neck pain. Please read this information carefully before deciding whether to participate.

## Purpose of the study

The purpose of this study is to describe and standardise the assessment of adherence to prescribed home-based therapeutic exercise using the Exercise Adherence Rating Scale (EARS). The study does not test a new treatment and does not require randomisation or allocation to different treatment groups.

## Why you are being invited

You are being invited because you are an adult receiving physiotherapy or rehabilitation after whiplash-associated disorder or non-surgical post-traumatic neck pain, and because a home exercise programme has been prescribed as part of your usual care.

## Voluntary participation

Participation is entirely voluntary. You may decide not to participate, or you may withdraw at any time without giving a reason. Your decision will not affect the care you receive.

## What participation involves

- You will be asked to provide basic clinical and sociodemographic information relevant to the study.
- Your prescribed home exercise programme will be documented using a structured record.
- After four weeks of home exercise prescription, you will be asked to complete the Exercise Adherence Rating Scale.
- Completing the questionnaire is expected to take approximately 5 minutes.
- No experimental treatment, biological sample collection, imaging procedure, audio recording or video recording is required by this protocol.

### Potential risks or discomforts

The study involves completion of questionnaires and collection of clinical information. No direct physical risk is expected from participation in the research procedures. Some participants may feel uncomfortable answering questions about adherence; they may skip questions or withdraw if they wish.

### Potential benefits

There may be no direct personal benefit from participating. The information obtained may help clinicians and researchers better understand adherence to home exercise after post-traumatic neck pain and may improve future rehabilitation assessment procedures.

### Costs and compensation

Participation will not involve any cost to you. No payment or financial compensation is planned for participation.

### Confidentiality and data protection

Your data will be coded using an alphanumeric participant code. Personal identifying information will be stored separately from the research database. Only authorised members of the research team will have access to identifiable data. Results will be reported in aggregate form so that individual participants cannot be identified.

### Use and publication of results

The results of this research may be published in scientific journals or presented at scientific meetings. Published results will not include information that identifies individual participants.

### Withdrawal from the study

You may withdraw your consent at any time by contacting the research team. You may also request information about your rights regarding access, rectification, erasure or restriction of your personal data, according to applicable data protection regulations.

### Contact details

For questions about the study or to exercise your rights, please contact: Daniel Pabón Carrasco, dpabon@us.es.

### Principal investigator / authorised researcher

__________________________________________ Date: ____ / ____ / ______

# File S2. INFORMED CONSENT FORM FOR PARTICIPATION IN THE RESEARCH STUDY

**Draft template for manuscript supplementary material. This document must be reviewed and approved by the corresponding Research Ethics Committee before use with participants.**

**Study title:** Assessment of Home Exercise Adherence in Adults with Whiplash-Associated Disorders and Non-Surgical Post-Traumatic Neck Pain: A Protocol Study Using the Exercise Adherence Rating Scale

**Participant name:** ______________________________________________

**Identification document number:** ______________________________________________

**Declaration of the participant**

Please tick each box if you agree with the statement.

☐ I have received and read the participant information sheet for this study.

☐ I have had the opportunity to ask questions and my questions have been answered satisfactorily.

☐ I understand the purpose of the study and what my participation involves.

☐ I understand that participation is voluntary and that I may withdraw at any time without giving a reason.

☐ I understand that refusal to participate or withdrawal from the study will not affect my clinical care.

☐ I understand that my data will be treated confidentially and coded for research purposes.

☐ I understand that the results may be published or presented for scientific purposes, without identifying me personally.

☐ I agree to participate in this research study.

**Participant signature**

__________________________________________ Date: ____ / ____ / ______

**Researcher obtaining consent**

__________________________________________ Date: ____ / ____ / ______

# File S3. PERSONAL DATA PROCESSING CONSENT FORM

**Draft template for manuscript supplementary material. This document must be reviewed and approved by the corresponding Research Ethics Committee before use with participants.**

**Study title:** Assessment of Home Exercise Adherence in Adults with Whiplash-Associated Disorders and Non-Surgical Post-Traumatic Neck Pain: A Protocol Study Using the Exercise Adherence Rating Scale

**Participant name:** ______________________________________________

## Personal data processing

I declare that I have read and understood the information about how my personal data will be processed for this research study. I understand that my personal data will be processed for scientific research purposes related to the assessment of adherence to prescribed home exercise after whiplash-associated disorders or non-surgical post-traumatic neck pain.

## Purpose of data processing

The purpose of data processing is to manage participation in the study, document eligibility and clinical context, record the prescribed home exercise programme, administer the Exercise Adherence Rating Scale and analyse adherence-related variables in coded form.

## Type of data processed

- Basic sociodemographic data, such as age and sex.
- Clinical data related to post-traumatic neck pain and rehabilitation.
- Data related to the prescribed home exercise programme.
- Questionnaire responses related to exercise adherence.
- No biometric data, audio recordings, video recordings or photographs are planned in this protocol.

☐ I agree and consent to the processing of my personal data for the scientific research purposes described above and in the participant information sheet.

☐ I understand that I may withdraw this consent according to applicable data protection regulations.

**Participant signature**

__________________________________________ Date: ____ / ____ / ______

**Researcher providing information**

__________________________________________ Date: ____ / ____ / ______

# File S4. CONSENT WITHDRAWAL FORM

**Draft template for manuscript supplementary material. This document must be reviewed and approved by the corresponding Research Ethics Committee before use with participants.**

**Study title:** Assessment of Home Exercise Adherence in Adults with Whiplash-Associated Disorders and Non-Surgical Post-Traumatic Neck Pain: A Protocol Study Using the Exercise Adherence Rating Scale

**Principal investigator:** José Antonio Martínez Fernández

**Study site:** Outpatient physiotherapy and rehabilitation clinics affiliated with or collaborating with the University of Seville, Seville, Spain

**Participant name:** ______________________________________________

**Participant code, if known:** ______________________________________________

## Withdrawal statement

I hereby inform the research team that I wish to withdraw my consent to participate in this study. I understand that withdrawal will not affect my clinical care.

Reason for withdrawal (optional):

________________________________________________________________________________
________________________________________________________________________________

## Scope of withdrawal

☐ I withdraw from any further participation in the study.

☐ I also withdraw my consent for future processing of my personal data where legally and technically possible.

☐ I request to be contacted by the research team to clarify the scope of my withdrawal.

**Participant signature**

__________________________________________ Date: ____ / ____ / ______

**Researcher receiving withdrawal**

__________________________________________ Date: ____ / ____ / ______

# Table S1. CLINICAL DATA COLLECTION SHEET

**Draft template for manuscript supplementary material. This document must be reviewed and approved by the corresponding Research Ethics Committee before use with participants.**

**Study title:** Assessment of Home Exercise Adherence in Adults with Whiplash-Associated Disorders and Non-Surgical Post-Traumatic Neck Pain: A Protocol Study Using the Exercise Adherence Rating Scale

**Participant code:** ______________________________________________

**Date of assessment:** ______________________________________________

**Core mandatory variables**

| Variable | Type | Unit / Coding | Value |
|---|---|---|---|
| Age | Quantitative | years | |
| Sex | Categorical | male/female/other/prefer not to say | |
| Clinical category | Categorical | WAD I, WAD II, non-surgical post-traumatic neck pain | |
| Time since trauma | Quantitative | weeks | |
| Mechanism of injury | Categorical | motor vehicle collision / fall / sports / other | |
| Pain intensity | Quantitative | 0-10 numerical rating scale or 0-100 mm VAS | |
| Current rehabilitation phase | Categorical | early/subacute/persistent; define according to protocol | |
| Number of supervised sessions completed | Quantitative | number | |
| EARS total score | Quantitative | 0-24 | |
| EARS completion time | Quantitative | minutes | |
| Missing EARS items | Quantitative | number | |

**Optional contextual variables**

- Medication use relevant to pain or rehabilitation.
- Comorbidities that may affect home exercise adherence.
- Self-perceived barriers to exercise if complementary EARS sections are used.
- Use of digital tools, videos, apps or printed handouts.

# Table S2. HOME EXERCISE PROGRAMME RECORD

**Draft template for manuscript supplementary material. This document must be reviewed and approved by the corresponding Research Ethics Committee before use with participants.**

**Study title:** Assessment of Home Exercise Adherence in Adults with Whiplash-Associated Disorders and Non-Surgical Post-Traumatic Neck Pain: A Protocol Study Using the Exercise Adherence Rating Scale

**Participant code:** ______________________________________________

**Date of prescription:** ______________________________________________

**Prescribing clinician:** ______________________________________________

**Programme documentation**

| Item | Required information | Value |
|---|---|---|
| Exercise type | mobility / motor control / strengthening / stretching / aerobic / mixed | |
| Number of exercises | total number prescribed | |
| Frequency | sessions per week | |
| Estimated duration | minutes per session | |
| Dose | sets, repetitions, hold time or time-based prescription | |
| Progression criteria | criteria for progression or modification | |
| Instruction format | verbal explanation / demonstration / written handout / video / app / other | |
| Individualisation | standardised / partially individualised / fully individualised | |
| Review schedule | planned follow-up or revision date | |
| Patient comprehension checked | yes / no; method used | |
| Accessibility needs | visual, hearing, cognitive or digital-literacy adaptations if relevant | |

**Clinician notes**

________________________________________________________________________________

________________________________________________________________________________

________________________________________________________________________________
